# β-adrenergic stimulation augments transmural dispersion of repolarization via modulation of delayed rectifier currents I_Ks_ and I_Kr_ in the human ventricle

**DOI:** 10.1038/s41598-017-16218-3

**Published:** 2017-11-21

**Authors:** C. Kang, A. Badiceanu, J. A. Brennan, C. Gloschat, Y. Qiao, N. A. Trayanova, I. R. Efimov

**Affiliations:** 1 The George Washington University, Washington, DC USA; 2 Washington University in St. Louis, St. Louis, MO USA; 3 John Hopkins University, Baltimore, MD USA

## Abstract

Long QT syndrome (LQTS) is an inherited or drug induced condition associated with delayed repolarization and sudden cardiac death. The cardiac potassium channel, I_Kr_, and the adrenergic-sensitive cardiac potassium current, I_Ks_, are two primary contributors to cardiac repolarization. This study aimed to elucidate the role of β-adrenergic (β-AR) stimulation in mediating the contributions of I_Kr_ and I_Ks_ to repolarizing the human left ventricle (n = 18). Optical mapping was used to measure action potential durations (APDs) in the presence of the I_Ks_ blocker JNJ-303 and the I_Kr_ blocker E-4031. We found that JNJ-303 alone did not increase APD. However, under isoprenaline (ISO), both the application of JNJ-303 and additional E-4031 significantly increased APD. With JNJ-303, ISO decreased APD significantly more in the epicardium as compared to the endocardium, with subsequent application E-4031 increasing mid- and endocardial APD80 more significantly than in the epicardium. We found that β-AR stimulation significantly augmented the effect of I_Ks_ blocker JNJ-303, in contrast to the reduced effect of I_Kr_ blocker E-4031. We also observed synergistic augmentation of transmural repolarization gradient by the combination of ISO and E-4031. Our results suggest β-AR-mediated increase of transmural dispersion of repolarization, which could pose arrhythmogenic risk in LQTS patients.

## Introduction

The QT interval is the time elapsed between ventricular depolarization and repolarization. A prolonged QT interval, caused by either drugs or genetic mutations, is termed Long QT syndrome (LQTS). It is well established that LQTS significantly increases the risk of sudden cardiac death from polymorphic ventricular tachycardia (VT), known as Torsade de Pointes (TdP)^1^. Over 50% of all LQTS patients face high risk of sudden cardiac death from VT. There are two distinct delayed rectifier potassium currents, rapid (I_Kr_) and slow (I_Ks_), which are primarily responsible for cardiac repolarization^2^. A majority of heritable LQTS cases, such as LQT1, LQT5, and LQT11, are associated with mutations in the KCNQ1 gene, which encodes I_Ks_^3^ or constituents of the I_Ks_ macromolecular complex^4,5^. However, in large mammals, it has been shown in certain studies that I_Kr_ is actually the dominant delayed rectifier potassium current where I_Ks_ only contributes relatively little to repolarization. Therefore, the generally accepted notion that I_Ks_ plays the biggest role in contributing to LQTS is puzzling^6,7^. Previous studies have demonstrated that I_Ks_ expression is highly variable in mammalian cells. For instance, guinea pig myocytes possess significant I_Ks_ current^2,8–10^, but mouse, rat, canine, and rabbit myocytes have little to no I_ks_ current^11–13^. In human myocytes, though the I_Ks_ blockade does prolong the QT interval, the amplitude of I_Ks_ has been reportedly low in human ventricular myocytes^14–16^. To complicate the question of I_Ks_ contribution to repolarization in the human heart even further, researchers often face experimental difficulties in recording I_Ks_ due to the various methods of isolating myocytes from human tissue^17–19^. Overall, the apparent controversial evidence between many clinical and experimental studies raises concerns about our current understanding of the role of I_Ks_ in human repolarization.

In addition to our relatively limited understanding of the general contributions of I_Kr_ and I_Ks_ in human cardiac repolarization, the individual contributions of these two currents also still remains unclear. In guinea pig myocytes, these two cardiac potassium currents are dynamic and significantly depend on the type of adrenergic receptor stimulation^20^. Stimulation of β_1,2_-adrenergic receptors is known to significantly augment I_Ks_^9,21–23^, whereas stimulation of β_3_-AR actually decreases I_Ks_^10^. Experimental evidence from guinea pig myocytes further suggests that the dominant repolarization current shifts from I_Kr_ to I_Ks_ during beta-adrenergic stimulation^20^. It remains unclear if this shift can be replicated in other mammalian models, such as humans, which have lower expression and thus a smaller contribution of I_Ks_. Recently, we have shown that I_Kr_ is significantly reduced in humans with end stage heart failure (HF), thereby indicating the possible increased significance of I_Ks_^24^.

These numerous uncertainties and continued controversies pose a significant hurdle for computational models of mammalian, and especially human, action potentials. The three most frequently used models of human ventricular myocyte action potentials, ten Tusscher^25^, Grandi^26^, and O’Hara-Rudy^27^, all behave differently under I_Ks_ or I_Kr_ blockade. Only the most recent O’Hara-Rudy model appears to most closely correlate with experimental data describing partial I_Kr_ block, yet early afterdepolarizations are present in the model and not observed in cardiac tissue experiments^28^. These discrepancies are detrimental to accurate predictions of arrhythmic risks. Following these lines of inquiry, we pursued a pharmacological investigation in the coronary perfused human left ventricular (LV) wedge preparation to examine the basal transmural contribution of I_Ks_ to repolarization, β-AR modulation of I_Ks_ and I_Kr_, and changes in transmural dispersion of repolarization due to β-AR stimulation and potassium channel block. Finally, we adapted electrophysiological conditions into a computational human myocyte model to corroborate our experimental results with computational ones.

## Methods

### Donor heart procurement

Adult donor human hearts were procured from either Washington Regional Transplant Community (WRTC) in Washington, DC or from Mid-America Transplant Services (MTS) in St. Louis, MO. Experimental and tissue procurement protocols were approved by the Institutional Review Boards at The George Washington University in Washington, DC and Washington University in St. Louis, MO. Heart tissue was de-identified by the organ procurement organizations prior to being provided to the research team. Methods and protocols described in these studies were performed in accordance with federal human research guidelines. A complete list of hearts with body mass index (BMI), cause of death, and LV ejection fraction (LVEF, listed when available) used in study are presented in Table 1. In this study, we completely excluded any donor hearts from victims of sudden cardiac death. Anoxia refers to anoxia of the brain.

**Table 1:** Donor Heart Information.

Organization	Age	Gender	BMI	Cause of Death	LVEF
MTS	38	M	18.2	Head Trauma	65%
MTS	63	F	22.51	CVA/Stroke	60%
MTS	19	M	30.42	Head Trauma	N/A
MTS	68	M	20.81	Head Trauma	N/A
MTS	68	M	18.13	CVA/Stroke	N/A
MTS	40	M	31.29	CVA/Stroke	N/A
MTS	59	F	19.92	CVA/Stroke	N/A
MTS	44	F	68.89	Anoxia	N/A
WRTC	76	M	45.6	CVA/ICH/Stroke	N/A
WRTC	57	M	28.4	CVA/ICH/Stroke	50–55%
WRTC	47	M	30.6	Anoxia	60%
WRTC	60	M	22.8	CVA/ICH/Stroke	N/A
WRTC	78	M	24.8	CVA/ICH/Stroke	N/A
WRTC	57	F	39.8	Anoxia	60–65%
WRTC	50	M	31.1	Anoxia	45%
WRTC	59	M	34.1	CVA/Stroke	N/A
WRTC	62	F	41	CVA/Stroke	65%

Hearts were acquired from Mid-America Transplant Services (MTS) and Washington Regional Transplant Community (WRTC). Age, gender, body mass index, and cause of death are listed for all hearts used in the study. Left ventricular ejection fraction (LVEF) was provided when echocardiography was performed and made available for transplant evaluation. Groups for separate protocols were not categorized with donor information provided. CVA: cerebrovascular accident; ICH: intracerebral hemorrhage. Anoxia refers to brain anoxia. No donor hearts from victims of sudden cardiac death were included.

Prior to removal from the chest, explanted donor hearts procured by either WRTC or MTS were cardioplegically arrested after aortic cross-clamp through systematic perfusion. MTS hearts were transported (15–20 minutes) to the laboratory in ice cold high potassium solution (in mmol/L: NaCl 110, CaCl_2_ 1.2, KCl 16, MgCl_2_ 16, NaHCO_3_ 10) to preserve the tissue. WRTC hearts were delivered approximately 2–3 hours post cross-clamp via courier in ice-cold commercial transplant preservation solution (Belzer UW) in the same manner as standard heart transplants. All hearts were categorized as non-failing donor hearts in line with all previously published data from our laboartory^29–31^.

### Experimental preparation

**Figure 1:**
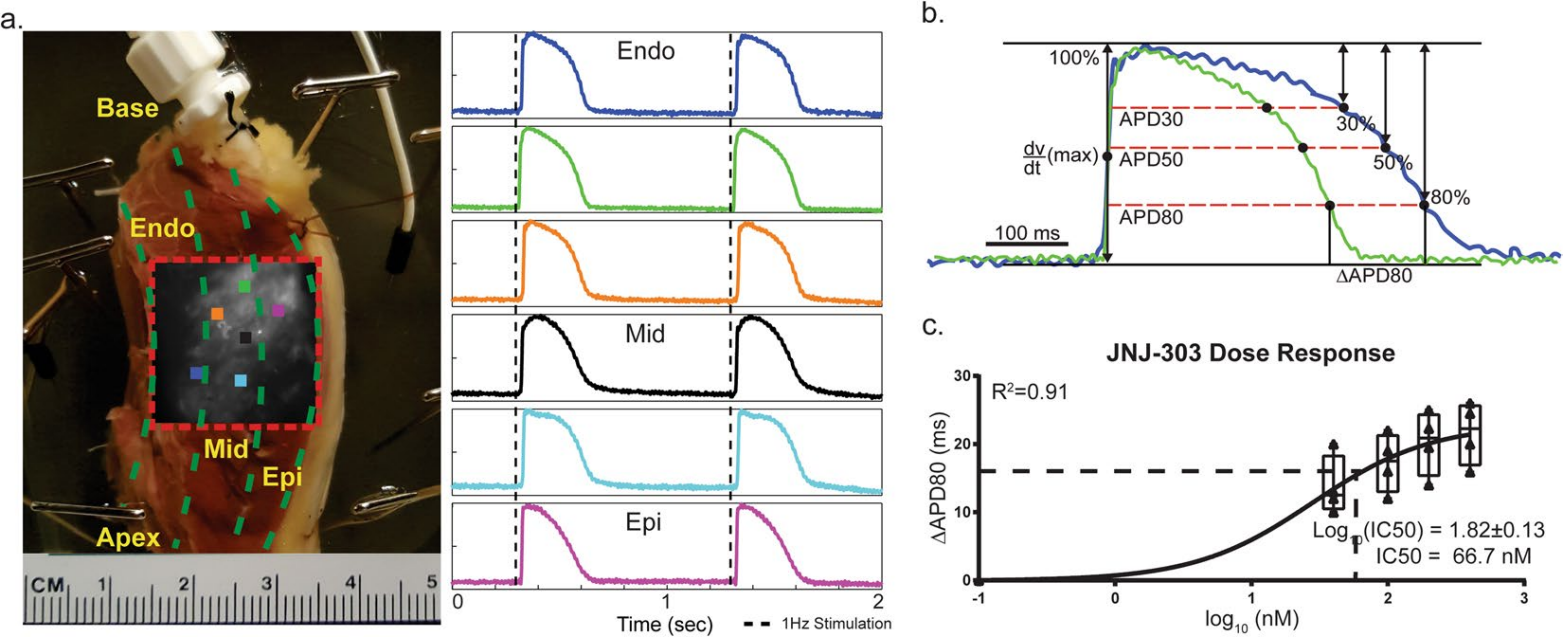
Experimental Preparations. Section of the left ventricular marginal area from a donor human hearts was dissected into a coronary perfused wedge preparation. (**a**) Orientation of the preparation was labeled accordingly. Color squares indicate recordings sites of the representative optical traces, illustrating that the tissue was fully perfused on the transmural surface. (**b**) Representative traces of how different repolarization % changes APD and ∆APD between conditions were calculated. (**c**) A third order sigmoidal curve was fitted to ∆APD80 vs Log (nM) for the dose response of JNJ-303. Log(IC50) was obtained as 1.82 ± 0.13 (±S.E.). 95% confidence interval for IC50 in nM was between 35.4 and 128.4 nM. Best fit value was determined to be 66.7 nM.

A coronary perfused section (wedge) from the marginal area of the LV (Fig. 1a) was dissected, cannulated, and electrically recovered as previously described^30^. The LV wedge was perfused with 37 °C oxygenated Tyrode’s solution in a 3D printed tissue chamber during experimentation. Constant coronary flow pressure of ~60 mmHg was carefully maintained. The wedge preparation was washed with 2L of Tyrode’s solution to remove any floating fat tissue or excess transplant solution, as well as to restore basal electrophysiology (EP)^31^ conditions prior to beginning dye loading. Between 5–10 µM of the excitation-contraction uncoupler blebbistatin was loaded into the tissue and allowed to perfuse for 15 minutes to stop contractions. Then, 3–5 µM of a transmembrane potential sensitive dye, di-4-ANEPPS, was loaded into the tissue through an injection port. A thin slice of the transmural wall was removed following blebbistatin and dye loading to improve signal quality. Two sensing needle electrodes were placed on either side of the wedge preparation, and one grounding electrode was placed inside the perfusion chamber. A pseudo-ECG was recorded with Powerlab 26T (AD instruments) throughout the duration of the experiment (Supplemental Fig. 3). A platinum-iridium tipped bipolar pacing electrode was placed on the transmural surface within the field of view to accurately assess both longitudinal and transverse conduction. Optical action potentials were mapped from an approximately 1.5–2.0 cm × 1.5–2.0 cm field of view at the transmural surface of the wedge (Fig. 1a) using a MiCAM Ultima L CMOS camera with high spatial and temporal resolutions (100 × 100 pixels, 1,000 frames/sec; SciMedia, CA).

### Electrophysiology experimental protocol

LV wedge preparations were studied under 3 different experimental protocols, labeled A, B, and C in Supplemental Fig. 1. Hearts procured from MTS were randomly selected for either a dose response protocol (n = 4, protocol A), or an I_Ks_ block protocol (n = 5, protocol B). Hearts procured from WRTC were used for a β-AR modulation of I_Ks_ and I_Kr_ block (n = 9, protocol C). In protocol C, the drugs were added at each step without washout, retaining the effect of previous agents. We applied a steady-state (S1-S1) restitution pacing protocol at twice the diastolic pacing threshold intensity to determine dependence of AP duration (APD) on pacing cycle length (PCL), which was progressively shortened from PCL = 2000 ms at 500, 200, and 100 ms intervals until the functional refractory period was reached. ISO (100 nM, Sigma Aldrich) was used for β-AR stimulation. JNJ-303 (200 nM, Sigma Aldrich) and E-4031 (1 µM, Sigma Aldrich) were used to selectively block I_Ks_ and I_Kr_, respectively. In a study published previously, the tissue retains its EP properties for up to three hours while in an experimental bath^32^.

### Data processing

**Figure 2:**
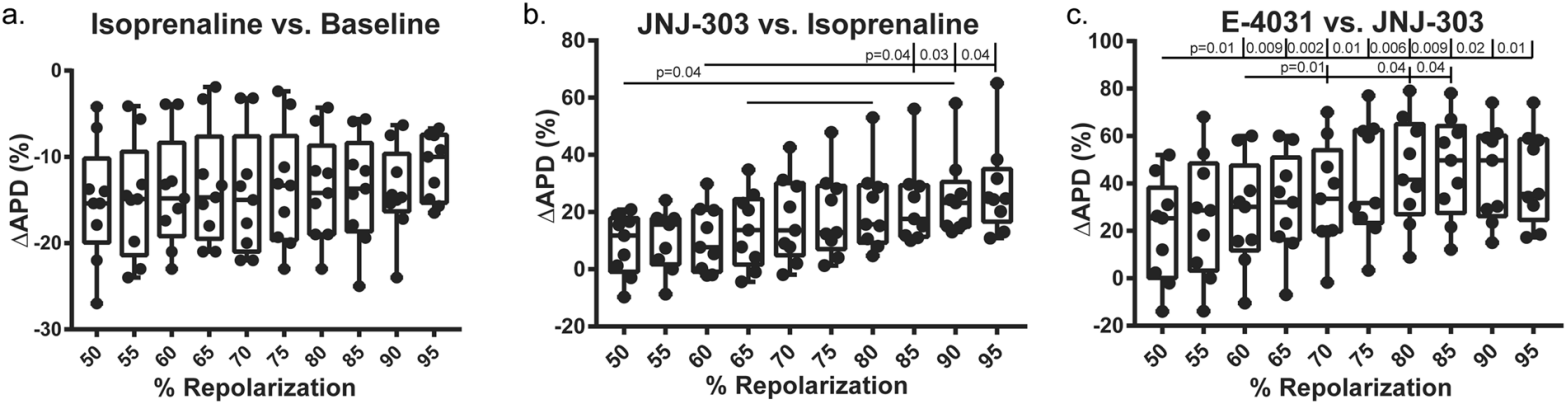
Percent Repolarization. Different pharmacologic interventions targeting specific ion channels led to changes in APD morphology. (**a**) Box-and-whisker (B&W) plots of % ∆APD80 from baseline to isoprenaline indicate that APD did not alter among different % repolarization. (**b**) I_Ks_ blockade with JNJ-303 linearly increased % ∆APD80 as % repolarization increased, indicating that I_Ks_ was more prominent at the latter phases of AP. (**c**) I_Kr_ block with E-4031 increased % ∆APD80 as % repolarization was increased, peaking at approximately 80–85% to indicate that I_Kr_ contributes the most at 80–85% repolarization.

**Figure 3:**
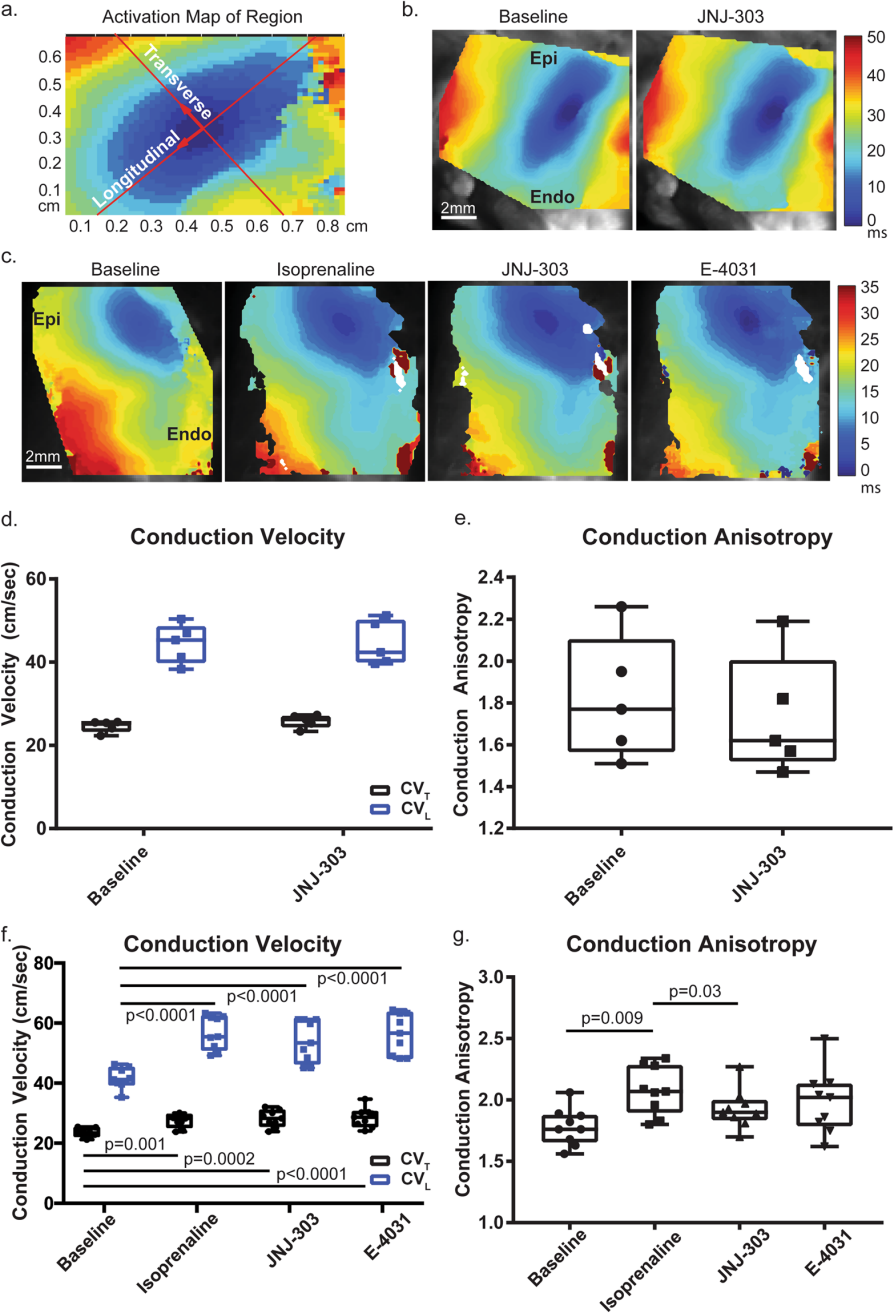
Conduction Properties. Conduction velocity (CV) was calculated from the transmural surface of the preparation. (**a**) A typical elliptical activation pattern was observed. Directions of activation (longitudinal, transverse) indicate the fastest and slowest conduction, respectively. (**b**) A representative activation map from protocol B shows that the activation pattern was the same both before and after application of JNJ-303. (**c**) Representative activation maps from protocol C indicate that conduction was increased with the application of isoprenaline. This change in activation was maintained with the addition of JNJ-303 and E-4031. (**d**) No significant difference was observed between transverse and longitudinal conduction velocities (CV_T_, CV_L_) in protocol B, and the anisotropy ratio was not altered (**d**). (**f**) Both CV_T_ and CV_L_ increased significantly with isoprenaline, but no other significant changes from previous conditions were observed. However, significant differences to the baseline were apparent. (**g**) Conduction anisotropy increased with isoprenaline, slightly decreased with JNJ-303, and remained unchanged with E-4031.

Rhythm, a custom-made MATLAB program designed for optical mapping data analysis, was used for calculating APD and plotting both activation and APD maps^33^. APs were filtered in space (3 × 3 pixel neighborhood) and time (low pass Butterworth filter at 100–150 Hz). Baseline fluorescent drift was removed with a first- or second-order fitted curve, as necessary. Activation times were determined by dV/dt_max_ and used to reconstruct activation maps and calculate conduction velocity (CV). APDs were calculated by measuring the elapsed time between activation and various repolarization percentages, as shown in Fig. 1b. For ease of comparison to previously published data from the human ventricular wedge^24,30,31^, as well as drug effectiveness (Fig. 2), we used APD measured at 80% repolarization (APD80). Both APD80 and CV were collected and analyzed at the physiological stimulation frequency of 1 Hz. ORCA, a custom MATLAB analysis package^34^, was modified and used for measurements of CV in the longitudinal and transverse directions (Fig. 3a). The directions were determined by the fastest and slowest CV from the pacing site (Supplemental Fig. 2).

### Statistics analysis

Statistical analysis was performed using Prism 7 (GraphPad). Box-and-whisker plots with all data points presented were used for all quantitative comparisons. Significant differences were labeled with individual p-values. Three different statistical tests were used in this study: Welch’s t-test, repeated measures of one-way ANOVA with post hoc Tukey’s honest significant difference for multiple comparisons (*RM 1way ANOVA THSD*), and two-way ANOVA with post hoc Tukey’s honest significant difference for multiple comparisons (*2way ANOVA THSD*). Different tests were used as necessary and as indicated in the results section.

### Computational simulations

The O’Hara-Rudy model of the human ventricular AP was used to perform computer simulations illustrating the EP behavior of a single cardiomyocyte under a protocol similar to the experimental one described above^27^. Similar to the model presented in Heijman *et al*., the model used in this study was adjusted to account for the protein kinase A (PKA) phosphorylation of eight different targets: ryanodine receptor, L-type calcium channel, phospholamban, I_Ks_, I_Na_, I_NaK_, I_Kur_, and troponin I, under both basal and β-stimulation conditions^35^. PKA phosphorylation was modeled as a binary process, where the given substrate was either considered phosphorylated or not phosphorylated. Therefore, the eight targets were divided into two subpopulations. At each time step, the current for a given substrate was computed by adding the currents passing through both phosphorylated and non-phosphorylated populations. The fraction of molecules allocated to each population was chosen so that the APs obtained from the simulations matched the ones recorded experimentally for both basal and β-AR stimulation conditions. The effects of JNJ-303 and E-4031 were modeled by setting the current through 80% of I_Ks_ channels and 20% of I_Kr_ channels equal to zero. The myocyte model was first paced at the physiological rate used in experimental tests (1 Hz) until steady state was reached, and the following 10 beats were recorded. APDs were computed for conditions like the experimental ones.

## Results

### Changes in APD at different degrees of repolarization

There are several definitions of APD used by experimentalists, with APD80 (APD at 80% of repolarization) being the most popular. In this study, we assessed how different pharmacologic interventions affected APD defined at different percentages of repolarization. Figure 2 shows changes in various APD percentages (from APD50 to APD95), caused by different pharmacologic interventions. β-AR stimulation shortened APD evenly across all APD percentages: from APD50 to APD95. ∆APD during β-AR stimulation was fitted by a linear curve with an R^2^ value of 0.62, and no significant difference in ΔAPD was observed among any repolarization percentages. JNJ-303 caused linearly increasing ∆APD (R^2^ = 0.97) as repolarization percentage increased. There were significant differences between lower and higher percentages of repolarization (50% vs. 90%, p = 0.04; 60% vs. 85%/90%/95% p = 0.04, p = 0.03, p = 0.04, respectively; *RM 1way ANOVA THSD*). Finally, ΔAPD vs. APD% caused by E4301 had a bell curve shape with a maximum value at ~APD80 to APD85. An exponential curve was used to fit this change (R^2^ = 0.15). Significant differences were observed between 50% and 60 to 95% repolarization (p = 0.01, p = 0.009, p = 0.002, p = 0.01, p = 0.006, p = 0.009, p = 0.02, p = 0.01; *RM 1way ANOVA THSD*), as well as between 60 and 70/80/85% (p = 0.01, p = 0.04, p = 0.04, respectively; *RM 1way ANOVA THSD*).

### β-AR stimulation significantly alters conduction properties

CV was calculated from protocols B and C with three pharmacological agents. The LV transmural surface exhibited a baseline longitudinal conduction velocity (CV_L_) of 44.4 ± 2.1 cm/sec and 41.6 ± 1.1 cm/sec and a transverse conduction velocity (CV_T_) of 24.6 ± 0.6 cm/sec and 23.5 ± 0.5 cm/sec, in protocols B and C, respectively. Conduction anisotropy (CV_L_/CV_T_) was 1.8 ± 0.1 and 1.7 ± 0.1, respectively. No difference in CV was observed in hearts acquired from different donor organizations. CV_L_ of 44.5 ± 2.4 cm/sec and CV_T_ of 25.8 ± 0.7 cm/sec were observed after acute application of JNJ-303, which produced an anisotropy ratio of 1.7 ± 0.1. No significant differences were observed in either CV (p = 0.56, p = 0.99; *2way ANOVA THSD*) or anisotropy ratios (p = 0.64; *Welch’s t-test*) (Fig. 3d,e).

In protocol C, ISO significantly increased both CV_L_ and CV_T_ to 56.8 ± 1.8 cm/sec and 27.5 ± 0.7 cm/sec (p < 0.0001, p = 0.0012; *RM 1way ANOVA THSD*), respectively. This bidirectional increase of CV from baseline was mostly maintained through subsequent administration of JNJ-303 and E4031. CV_L_ and CV_T_ values after application of JNJ-303 were 54.1 ± 2.35 cm/sec and 28.2 ± 0.9 cm/sec, respectively. Both CV_L_ and CV_T_ remained unchanged after final application of E4031(56.3 ± 2.3 cm/sec and 28.6 ± 1.0 cm/sec, respectively). The ratio of anisotropy was increased to 2.1 ± 0.2 after ISO, indicating that the increase of CV_L_ exceeded that of CV_T_. This ratio was, however, significantly decreased to 1.9 ± 0.05 after JNJ-303 (p = 0.03, *RM 1way ANOVA THSD*). This decrease, though also observed in protocol B, did not reach statistical significance. Furthermore, additional application of E4031 did not significantly alter anisotropy (2.0 ± 0.08) (Fig. 3f,g).

### β-AR stimulation modulates I_Ks_ and I_Kr_

Subsequent APD analyses were based on results from previous sections. Our data showed that APD80 was indeed the optimal definition due to the effectiveness of measuring ∆APD80 for both I_Ks_ and I_Kr_. Furthermore, from protocol A, we determined that the log of IC50 for JNJ-303 in human ventricular tissue was 1.82 ± 0.13 (±S.E.) with 95% confidence interval for IC50 in nM between 35.4 and 128.4 nM (Fig. 1c). The best fit IC50 was 67 nM, which determined the saturation concentration of 200 nM which we used in protocols B and C.

**Figure 4:**
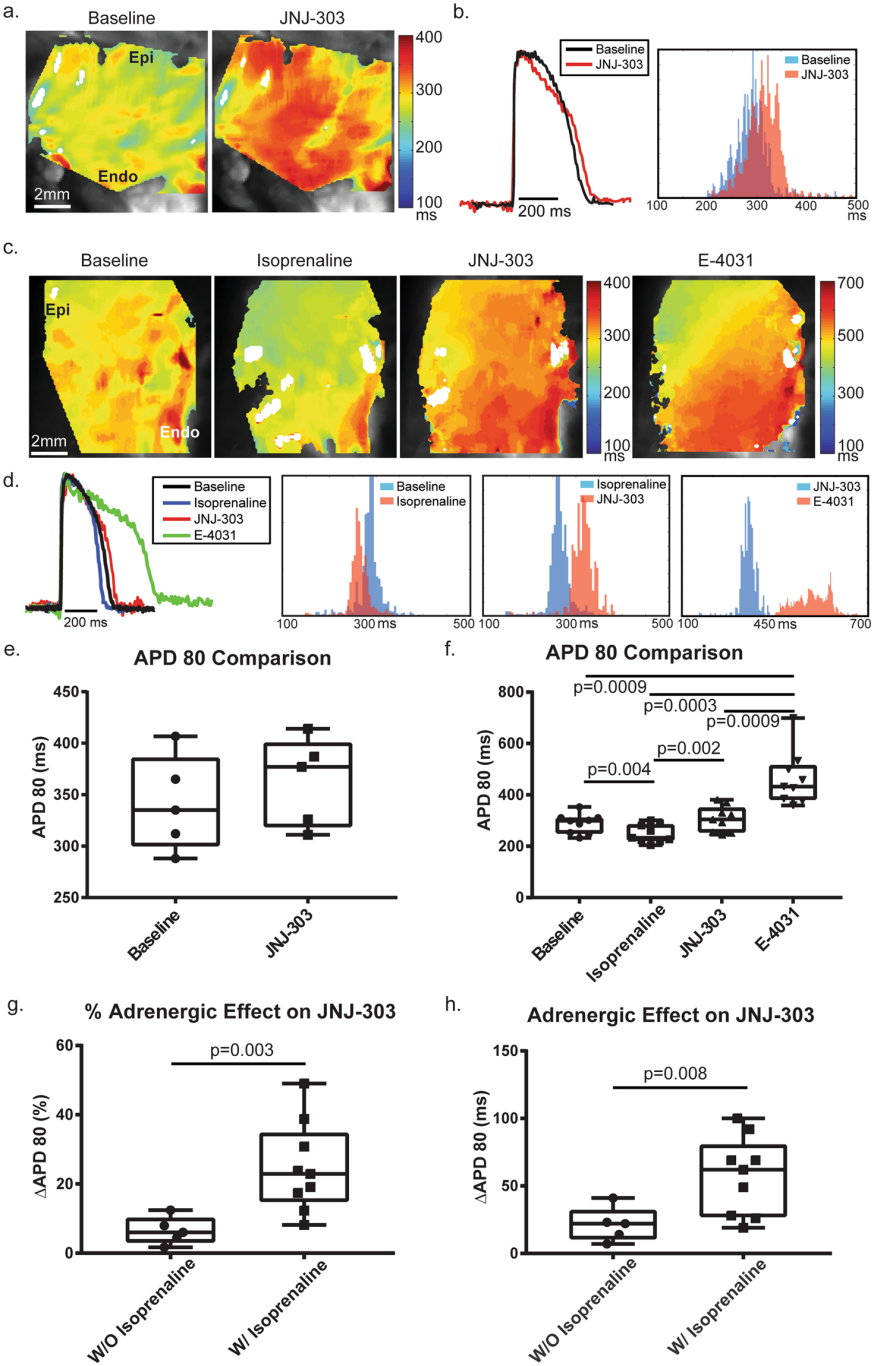
APD80 Comparisons. APD was measured at 80% of repolarization. (**a**) APD80 maps for both conditions in protocol B show that JNJ-303 increased APD80 slightly (but not significantly), as indicated by the red color. (**b**) Representative traces show changes in APD80 distribution after application of JNJ-303. Blue indicates baseline and red indicates the pharmacologic intervention. (**c**) APD80 maps for all conditions in protocol C highlight that isoprenaline decreased APD80, JNJ-303 increased APD80 above baseline, and E-4031 dramatically increased APD80. (**d**) Representative traces for each condition in protocol C show distribution histograms for three pairs of comparisons: baseline vs. isoprenaline, isoprenaline vs. JNJ-303, and JNJ-303 vs. E-4031. Bars in blue are representative of previous treatment, while bars in red are representative of post treatment. (**e**) B&W plots indicate a slight but not significant increase in APD80 with I_Ks_ block. (**f**) Significant reduction of APD80 was observed with application of isoprenaline, and an increase of APD80 was observed with both I_Ks_ and I_Kr_ block. (**g**,**h**) ∆APD80 in both ms and % of pre and post JNJ-303 with or without isoprenaline stimulated β-ARs. ∆APD80 drastically increased in both cases. See text for details.

Baseline APD80 for protocols A and B were 372 ± 16 ms and 342 ± 11 ms, respectively (averaged 354 ± 13), and 288 ± 13 ms (p = 0.0021; *Welch*’*s t-test*) for protocol C (Supplemental Fig. 7), indicating basal differences in donor hearts from the two different geographical regions in the United States. Previous reports from isolated rat myocytes showed that β-AR stimulation significantly increased I_Ks_ and its relative contribution to the repolarization process^20,21,36^. Our study indicated a similar trend (Fig. 4). Isolated JNJ-303 slightly increased the APD80 to 363 ± 19 ms ms, a 21.4 ± 5.7 ms ms (or 6.5 ± 1.8%) prolongation (p = 0.47, *Welch*’*s t-test*). One the other hand, in protocol C, ISO significantly reduced the APD80 to 249 ± 12 ms first (p = 0.004, *2way ANOVA THSD*). Subsequent addition of JNJ-303 dramatically increased the APD80 to 306 ± 17 ms (p = 0.002, *2way ANOVA THSD*), a 57.1 ± 9.6 ms (or 24.7 ± 4.3%) prolongation (p = 0.008 and p = 0.003; *Welch*’*s t-test*). The final application of E-4031 further increased the APD80 to 463 ± 35 ms, a 157 ± 24 ms (or 51.3 ± 6.6%) prolongation. This change by E-4031 was most statistically significant compared to all previous conditions (p = 0.0009, p = 0.0003, p = 0.0009, *2way ANOVA THSD*). Previously published data from our laboratory using the same preparation documented an increase of approximately 65% when E-4031 was applied without any β-AR stimulation^24^.

### Shifts in restitution properties

**Figure 5:**
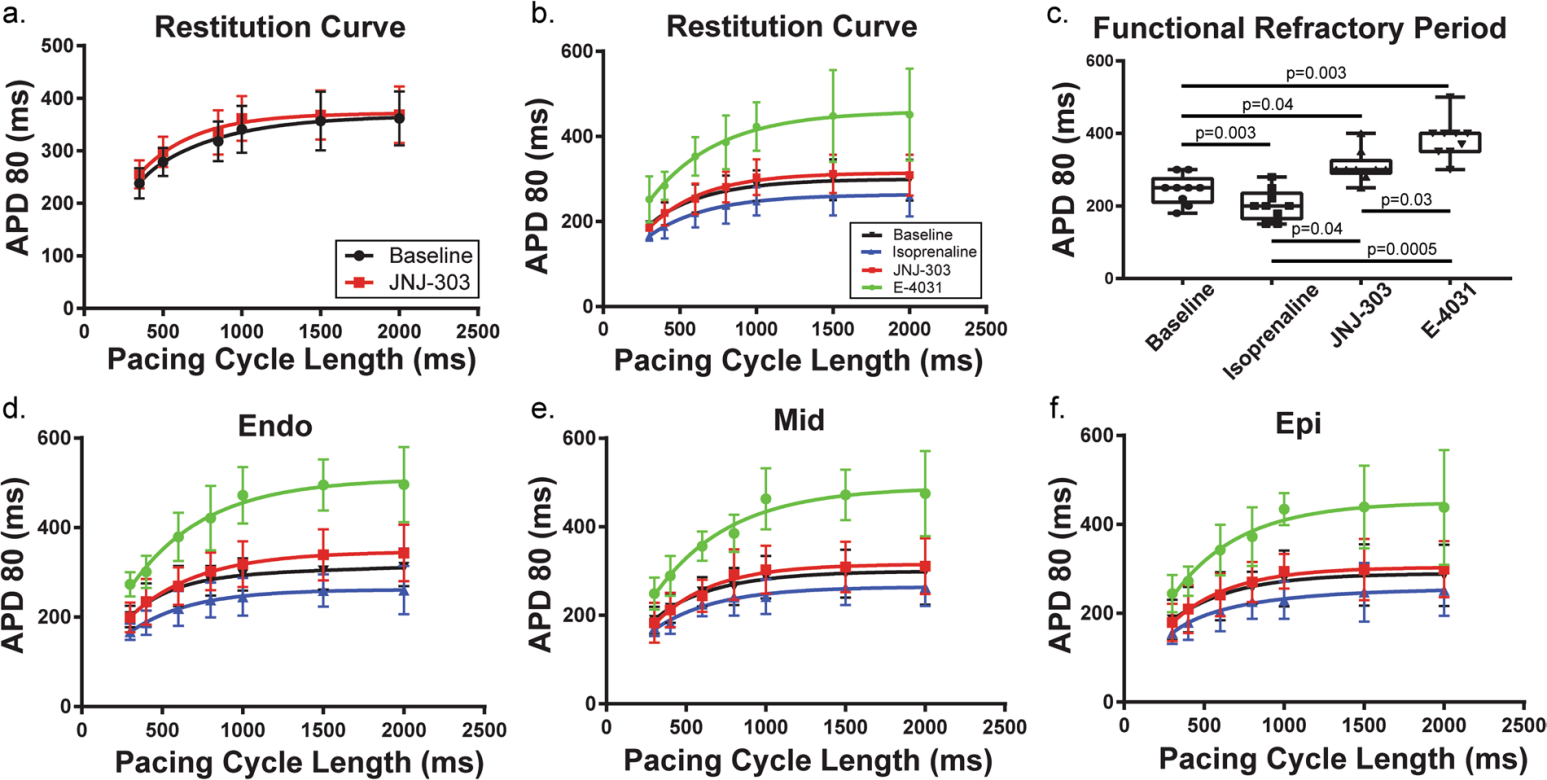
Restitution Properties. Using an S1S1 pacing protocol, restitution curves show that APD80 decreased as pacing cycle length decreased. This relationship was fitted using a two phase exponential curve. (**a**) The restitution curve shifted minimally with the addition of JNJ-303 in isolation. (**b**) Restitution downshifted with isoprenaline, upshifted with I_Ks_ block, and further upshifted with I_Kr_ block. (**c**) Functional refractory period was reached when S1S1 pacing lost capture. The refractory period significantly decreased with isoprenaline, and increased with both I_Ks_ and I_Kr_ blockers. (**d**–**f**) Restitution curves were plotted for endo, mid, and epi areas of the transmural surface indicating similar changes across all three areas.

APD restitution curves for each condition were fitted with a two-phase exponential curve starting from an S1–S1 of 300 ms to 2000 ms. In protocol B, the APD restitution curve was shifted slightly upward with the addition of JNJ-303. This upward shift brought the restitution curve above the baseline. In protocol C, the APD restitution curve was also shifted upward with the addition of JNJ-303, but it shifted downward with application of ISO. Addition of E-4031 dramatically shifted the curve upward. These same trends were observed when examining each transmural section separately (Fig. 5). The functional refractory period also changed in accordance with pharmacologic intervention (in ms): baseline 244 ± 13, ISO 203 ± 14, JNJ-303 309 ± 14, E-4031 385 ± 18. Significant differences were observed amongst all treatments (Fig. 5c).

### Transmural dispersion of repolarization was altered by pharmacologic interrogation

**Figure 6:**
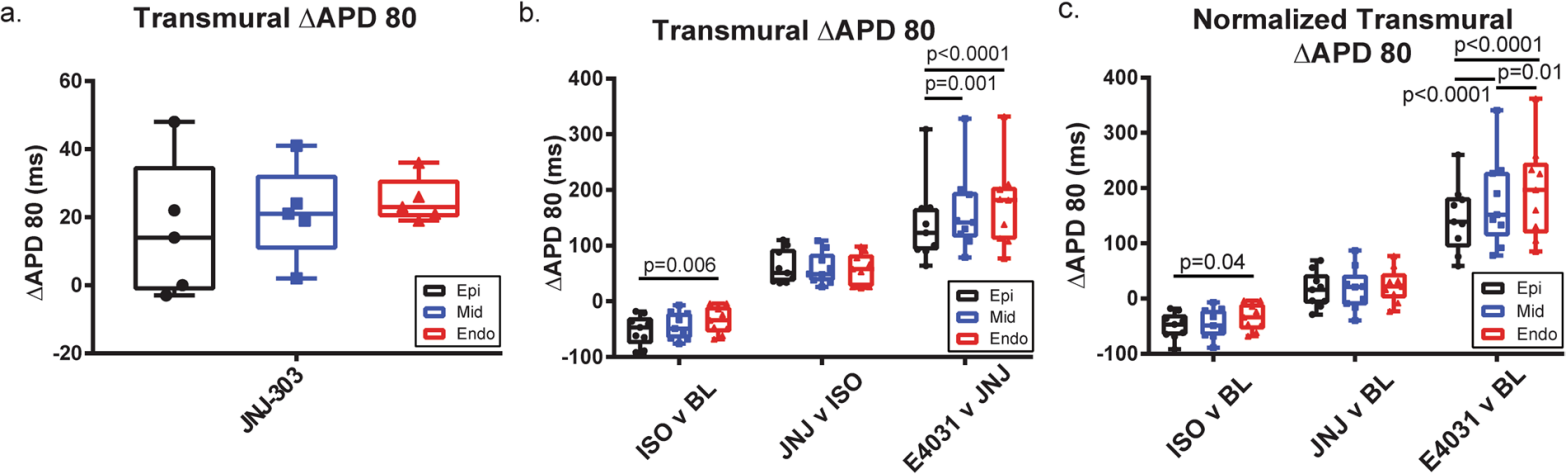
Transmural Dispersion. Absolute ∆APD80 was calculated in milliseconds from epi, mid, and endo areas of the transmural surface. (**a**) No significant difference was observed from ∆APD80 across the transmural surface post I_Ks_ blockade in protocol B. (**b**) ∆APD80 was calculated as the difference between post and pre-treatment. APD80 reduction from isoprenaline was significantly greater in the epicardium as compared to the endocardium. On the other hand, APD80 under I_Kr_ blockade increased more significantly in the midmyocardium and endocardium as compared to the epicardium. (**c**) Normalized ∆APD80 was calculated as the difference between post treatment and baseline. The transmural gradient was increased by both isoprenaline and E-4031, a trend which was also observed in comparing pre- and post-treatments.

Dispersion of repolarization across the transmural surface of the human LV was also characterized. APD80 maps and histograms (Fig. 4a–d) indicate that the APD increased from the epicardium to the endocardium. This gradient correlate to results already reported in both animal and human models. To quantitatively analyze the effects of pharmacologic intervention, the transmural surface was subdivided into three parts: endocardium, midmyocardium, and epicardium (Fig. 1a), similar to previous studies. Figure 6 shows ∆APD80 in ms (% values shown in S\Supplemental Fig. 5). The percentage change of APD80 was calculated by [(APD80_Cond2_ − APD80_Cond1_)/APD80_Cond1_] * 100. In protocol B, ∆APD80 increased from epi to endo both in ms and % (epi: 16.2 ± 9.2 ms/5.2 ± 2.9%; mid: 21.4 ± 6.2 ms/6.5 ± 1.9%; endo: 25 ± 3.0 ms/7.4 ± 0.9%). However, this trend was not significant. Notably though, drug effects on the epicardium had the greatest variance, and this variance decreased toward the endocardium.

Transmural ∆APD80 was shown first in protocol C (Fig. 6b). Here, ISO decreased APD more significantly in the epicardium as compared to the endocardium (epi: −52.3 ± 8.9 ms/−18.6 ± 2.9%; endo: −34.1 ± 8.1 ms/−11.1 ± 2.4%; P = 0.006, P = 0.008, respectively; *2way ANOVA THSD*). Midmyocardial ∆APD80 fell in between these two values but was not significantly different from either (−43.8 ± 8.2 ms/−14.9 ± 2.6%). After administration of JNJ-303, this difference was abolished (epi: 61.6 ± 10 ms/27.6 ± 5.3%; mid: 59.3 ± 9.5 ms/24.3 ± 4.3%; endo: 57.8 ± 9.4 ms/22.1 ± 3.6%). However, the administration of E-4031 produced a significant transmural gradient in ∆APD80. Both the midmyocardium and endocardium were significantly more affected as compared to the epicardium (epi: 139 ± 24 ms/46.5 ± 6.3%; mid: 161 ± 24 ms/53 ± 6.8%; endo: 172 ± 25 ms/53.3 ± 6.2%; p = 0.001, p < 0.0001; *2way ANOVA THSD*).

The transmural ∆APD80 was also evident after normalization to the baseline APD (Fig. 6c). Similar significant differences were maintained. As ISO was the first drug administered to baseline, the comparison remains the same. JNJ-303 again abolished transmural ∆APD80 gradient (epi, 15.8 ± 8 ms/6 ± 4.2%; mid, 16.7 ± 13 ms/6.5 ± 5.1%; endo 23.7 ± 10 ms/8.3 ± 3.9%). Finally, the application of E-4031 affected the midmyocardium and endocardium more than the epicardium (epi, 146 ± 21 ms/54.8 ± 8%; mid, 176 ± 27 ms/62.1 ± 8.9%; endo 195 ± 29 ms/66.1 ± 8.8%; p < 0.0001, p < 0.0001; *2way ANOVA THSD*). Interestingly, the endocardium also presented significantly increased % ∆APD80 when compared to the midmyocardium (p = 0.01; *2way ANOVA THSD*).

### Computational stimulation reflects experimental β-AR stimulation and I_Ks_ blockade

**Figure 7:**
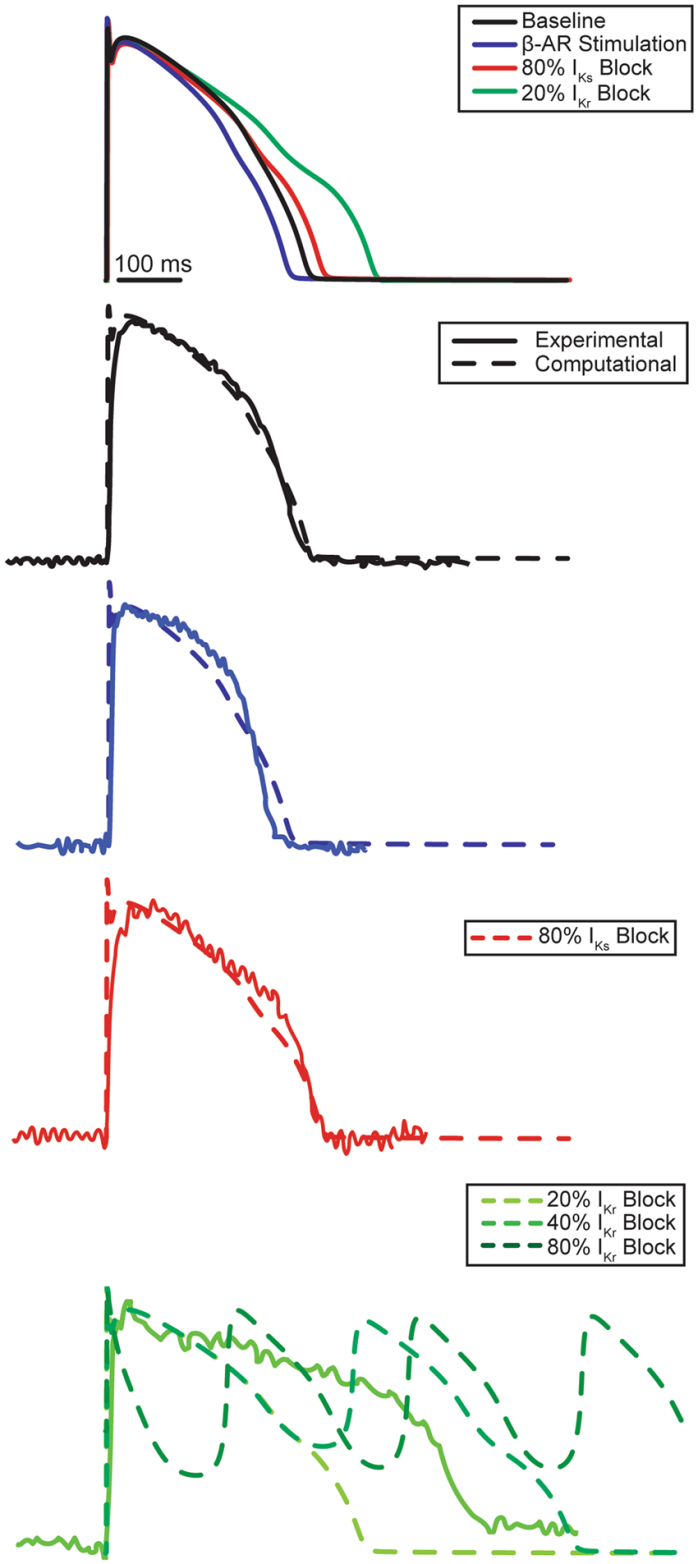
Computational Modeling of I_Ks_ and I_Kr_ Block with β-AR Stimulation. Protocol C was repeated in a computational model. The top panel displays AP traces obtained by sequential application of various pharmacologic interventions in the model. Baseline experimental AP traces were accurately reproduced by the model. β-AR stimulation was produced by an increased phosphorylation of all ion channels. The Model was also able to reproduce experimental data for I_Ks_ block at 80% current blockade. However, I_Kr_ blockade could not be reproduced using the modified O’Hara-Rudy model. When I_Kr_ block exceeded 20% early afterdepolarizations (EAD) were observed. EADs were not observed in any preparation even after saturated I_Kr_ block.

The computer model was calibrated to reproduce experimental APDs observed at the baseline and during β-AR stimulation (Fig. 7). The fractions of I_Ks_ and I_Kr_ channels phosphorylated by PKA during baseline conditions were set to 15%, while none of the other substrates were considered phosphorylated. During β-AR stimulation, all I_Ks_ and I_Kr_ channels and 25% of the other substrates were phosphorylated. The only exception was the L-type calcium channels, for which only 10% of the molecules were phosphorylated. This caused stimulated APD80 to shorten by 8.5%. After fixing these conditions, an 80% I_Ks_ block was applied to replicate an APD prolongation similar to that seen in experimental data, which is equivalent to a 14.6% increase in APD80. Experimental data for I_Kr_ could not be replicated due to occurrence of EADs following 20% or higher block (13.8% increase in APD80 from previous conditions).

## Discussion

### Summary of findings

For the first time, this study examined the role of β-AR stimulation in human LV repolarization, including modulation of its transmural dispersion. Our data indicates that the delayed rectifier repolarization potassium currents, I_K,_ may be strongly modulated by β-AR in humans. Under baseline conditions (in absence of β-AR stimulation), I_Kr_ is the predominant component of human LV repolarization. I_Ks_ is still present in the human LV, but it is relatively small. In this study, we observed that β-AR stimulation significantly increased the contribution of I_Ks_ to human LV repolarization. Moreover, combined β-AR stimulation and block of potassium channels resulted in significant transmural dispersion of repolarization, which is a known substrate for arrhythmias. Specifically, this study determined the acute effects of a specific I_Ks_ blocker, JNJ-303, on human LV repolarization, both with and without β-AR stimulation. At baseline conditions, JNJ-303 had no effect on CV (as expected), but it slightly increased APD80. β-AR stimulation significantly increased the activation of I_Ks_, which was evident from a far more dramatic prolongation of APD80 produced by the application of JNJ-303 while under the effects of ISO. However, I_Kr_ block by application of E-4031 had a lesser effect on APD80 as compared to our previously reported data without β-AR stimulation^24^, suggesting a reduction of I_Kr_ contribution to human LV repolarization during β-AR stimulation. Furthermore, we identified regional EP differences among donor hearts; hearts acquired in St. Louis exhibited significantly longer APD values as compared to hearts acquired in Washington DC, while no difference in CV was observed. This difference in the APD can be attributed to different institution’s surgical procedure and supplies, namely cardioplegia, as well as differences in transportation time from the operating room to the laboratory.

### β-AR stimulation increased tissue anisotropy

In healthy human hearts, β-AR receptors primarily activate G_s_ protein, which in turn, activates adenylyl cyclase pathway converting adenosine triphosphate (ATP) to cyclic adenosine monophosphate (cAMP). Increased cAMP concentrations lead to an increased activation of PKA. Through PKA phosphorylation of the principal gap junction protein connexin 43 (Cx43) and inward sodium channels (I_Na_), CV is increased. ISO, a non-selective β-AR agonist, effectively increased both CV_T_ and CV_L_, as well as the anisotropy ratio. In healthy tissues, Cx43 is localized at the ends of the myocytes, and lateralization of Cx43 is minimal^37^. I_Na_ also tends to localize to the cell ends^38^. The greater increase in longitudinal CV_L_ by β-AR stimulation can be explained by this localization.

### Choice of pharmacologic agents and concentration

The concentrations of drugs used in this study were comparable to those published previously. 1 µM E-4031 has shown to completely block I_Kr_ channels, and this amount was used in previous studies published by our laboratory^12,24^. Many I_Ks_ blockers have also been used in past studies; for example, benzopyran chromanol 293B and its more potent analogues (HMR-1556, L-768,673, and BMS-208,782) are frequently used in animal models^14,39^. Studies have concluded that these I_Ks_ blockers do not directly induce arrhythmias in the respective models^40^. However, in some models, the addition of I_Ks_ blockers in concentrations that usually do not affect hERG can still prolong the QT interval and reproducibly cause LQT1-type TdP with ISO^41^. JNJ-303, a more recently developed specific I_Ks_ blocker, has a significantly reduced effect on I_Kr_ as compared to other previously used agents. JNJ-303 has been reported to not markedly affect I_Kr_, prolong APD, nor induce EADs below a concentration of 10 µM^14^. The best fit IC_50_ from our human sample was approximately 67 nM, which is close to the previously reported 64 nM^14^. We selected 200 nM for comparison studies because it saturates the I_Ks_ blockade but is safe from triggering much more significant QT prolongation via binding to hERG.

### Percent repolarization reflects alteration of APD morphology

In accordance with their names, the rapid and slow rectifier potassium currents contribute to AP repolarization in a consecutive fashion. ISO activates PKA through the signaling cascade described above, which then phosphorylates several different ionic channels and pumps as evident from both an observed increase in CV and shortening of APD. Since PKA phosphorylation is not selective, % ∆APD does not vary with the definition of % repolarization at which the APD was measured. Being the slower component of the rectifying current, I_Ks_ predominately affects the latter portion of repolarization. The observed linear increase of % ∆APD across different % repolarizations corroborated this idea. Conversely, the rapid component, I_Kr_, most prominently affected % ∆APD at 80 to 85% repolarization before its effect dropped off at higher percentages of repolarization. These results are evident from the I_Kr_ blocker having the largest impact on APD80 to APD85, while the I_Ks_ blocker most affected APD95. With most recording methods, APD95 to APD100 are typically not reliable due to noise, thus APD80 to APD85 were determined to be the best definitions of APD for studies of human I_Ks_ and I_Kr_.

### Impact of increased I_Ks_ in the human left ventricle

It is well established that I_Kr_ and I_Ks_ both play major roles in cardiac repolarization of large mammals. However, expression and relative contribution of I_Ks_ varies from study to study and from animal model to model^28,39,42^. Similar to the results observed in canine and rabbit myocytes studies, the specific I_Ks_ blocker at a saturated specific binding concentration of 200 nM did not significantly increase AP duration. The increase of APD, while detectable, was relatively minor.

An increased response to I_Ks_ block or increased I_Ks_ after β-AR stimulation has been widely reported in canine and guinea pig myocytes^20–23^. Response in humans, however, has not been well documented, especially in intact tissue. Rate dependency of I_Ks_ and I_Kr_ deactivation has also been previously reported, yet this continues to be debated. Using restitution pacing, we found that APD prolongation in the human left ventricle was not dramatically altered in relation to the pacing rate throughout the transmural surface. This finding is similar to what has been found in canine tissue. Canine studies have shown that specific β_1,2_-AR stimulation increases I_Ks_, an observation of which has been further corroborated in guinea pig studies^36^. β_1_- and β _2_-AR are two G protein-coupled receptors in the heart which allow for the activation of various signaling pathways for diverse cellular responses. β_1_-AR primarily binds to G_s_, whereas β_2_-AR can bind to either G_i_ or G_s_ in healthy tissue, depending on the PKA-dependent phosphorylation. Therefore, the effect of β_2_-stimulation on I_Ks_ is dependent on PKA activity β_3_-AR is a third beta isoform which acutely activates a separate signaling pathway through the G_q_-protein and decreases I_Ks_^10^. Since there is a relatively low abundance β_3_-AR in the healthy human heart though, the non-specific β-AR agonist primarily activates the dominant isoform (β_1_-AR) which is coupled to the G_s_ protein. Still, the effect of isolated β_2_-stimulation of human I_Ks_ has yet to be elucidated. For this reason, it would be worthwhile to examine the effects of isolated β_1_- and β_2_-AR stimulation on I_Ks_, especially in considering the reversal of β_1_- versus β_2_-AR signaling during heart failure and concomitant slowing of repolarization^31,43^.

### β-AR stimulation alters contribution of delay rectifier potassium currents

The relative contribution of the two rectifier currents to repolarization has been debated. I_Kr_ is generally considered the dominant repolarizing current, yet mutations in I_Ks_-related genes are responsible for three of the most common LQTS cases: LQT1, LQT5, and LQT11^3,4,44^. Unfortunately, most published studies have only examined a single current at a time in isolated myocytes. Recently developed individual cell techniques, such as “onion-peeling” or “dynamic clamping”, can record multiple currents in the same cell^20,45–47^. Results of these new techniques are quite exciting. Studies of guinea pig repolarizing currents using the onion-peeling technique have suggested that different catecholamine levels alter the contribution of the two potassium currents. Under 30 nM of ISO, it was shown that I_Kr_ remains unchanged and I_Ks_ increases dramatically, far surpassing I_Kr_ in its relative contribution to repolarization^20^. As discussed in the previous section, our data shows that ISO increased the impact of the I_Ks_ blockade, indicating a greater contribution from I_Ks_. Our study also showed that the I_Kr_ blocker E-4031 had less of an effect after β-AR stimulation than what was observed in previously published data without such stimulation at the same drug concentration^24^. Thus, we report novel findings of a shift in balance of delayed rectifier potassium channels in human, results of which are critical to understanding causes of sudden cardiac death in LQT1 patients during beta-adrenergic stimulation.

### Enhanced transmural dispersion via β-stimulation and I_Kr_ blockade

Transmural dispersion of repolarization has been shown to be a powerful functional substrate for arrhythmogenesis. It has widely been reported in coronary perfused wedge studies of different animal and human models that APD increases from epi- to endocardium^30,31,42^. Under normal conditions, this dispersion of APD is small and benign. Our study examined how pharmacologic interrogation increases this dispersion and potentially contributes to a functional block. β-AR stimulation decreased APD more dramatically in the epicardial region as compared to the midmyocardium and endocardium. While the β_1_ receptor density is reportedly even across the transmural surface of normal hearts, it is known to decrease drastically in the endocardium during HF^48^. On the other hand, we observed that I_Kr_ block had a significantly stronger effect on APD in the endocardium as compared to the midmyocardium and even more so as compared to the epicardium. A transmural gradient expression of human I_Ks_ or I_Kr_ has yet to be reported, but it has been shown to be non-significant in canine tissues (without β-AR stimulation)^49^. In our study, β-AR stimulation reduced epicardial APD while I_Kr_ block increased endocardial APD, the combined effect of which would result in increased transmural dispersion of repolarization (Supplemental Fig. 8). This transmural gradient could provide the substrate for functional block and arrhythmias. It is important to note that I_Ks_ did not have a significant impact on the transmural gradient, with or without β-AR stimulation. Thus, it is more likely that the combination of an increased CV from β-AR stimulation and a prolonged APD from I_Ks_ block provide enough repolarization de-synchrony to trigger and sustain TdP in LQT1 patients.

Moreover, while enhanced transmural dispersion is pro-arrhythmic, increased wavelength is anti-arrhythmic, because it requires a much greater amount of tissue to sustain reentry^50,51^. Supplemental Fig. 6 shows that wavelength was increased with every pharmacologic intervention due to both increased CV and APD. Cardiac susceptibility to arrhythmias depends on the balance between wavelength and transmural heterogeneity.

### Computational stimulation reflects experimental β-AR stimulation and I_Ks_ blockade

The phosphorylation states before and after β-AR stimulation were adjusted to match experimental APs. Despite using a saturating dosage of the I_Ks_ blocker JNJ-303, computational simulation indicated that not all I_Ks_ channels were blocked. We suspect this was due to remaining problems with the O’Hara-Rudy computer model. For example, the model could only support a 20% I_Kr_ block prior to inducing EADs. This was in contrast to experimental data, where even a saturated I_Kr_ blockade did not produce any EADs. While this model was able to recapitulate experimental data obtained during I_Ks_ blockade relatively closely, it still failed to represent I_Kr_ block similar to the original O’Hara-Rudy model. This major difference is likely due to strong cell-cell coupling that is only present when considering tissue response^52^. Alternatively, other repolarizing currents, such as small conductance potassium currents, could be present, which are not currently incorporated in the O’Hara-Rudy computer model. A tissue model is currently being developed using presented experimental data.

### Advantages and limitations of a pharmacologic interrogation approach

In most experimental models, isolated whole-cell voltage clamp is the approach of choice when measuring ionic currents. However, it has been shown that for human tissue, which is highly fibrotic especially in less healthy donor hearts, a powerful cell isolation protocol is required. The isolation procedure can become harmful to membrane multiprotein complexes, including ionic currents. Alterations of an I_Ks_ multiprotein complex due to experimental cell isolation procedure is likely^19^. Additionally, the increase of CV from β-stimulation is crucial in the mechanism by which I_Ks_ can induce TdP. Therefore, the model of a coronary perfused wedge preparation represents a powerful approach. Furthermore, recently developed cardiac organotypic slice preparations can further extend these studies of human repolarization because of their abilities to recapitulate both acute and chronic effects of drugs (e.g. chronic α- and β-AR stimulation) in inducing repolarization remodeling^53^.

The pharmacologic agents used in this study were highly specific at the concentrations used, as already demonstrated by multiple other groups. However, I_Kr_ conductance is strongly dependent on other channels. Experimental protocols that involve a blockade of the different channels used here can alter their individual effects and were not necessarily fully specific to each of their targets. Therefore, it is important to consider complex context in data interpretation. Nevertheless, despite the lack of specificity of some pharmacologic agents, the magnitude of significance found in this study outweigh the limitations of the approach.

Finally, it is important to take into consideration the high variability of human subjects in this study, such as gender and age. Information on the exact health status of donor hearts was very limited. While most hearts were rejected for transplantation due to age restriction, there were unavoidable samples that had early onset of heart disease but were asymptotic. All available donor hearts (excluding those from cardiac death) were utilized due to the scarcity nature of tissue. Regardless, all hearts used in this study were categorized as non-failing hearts, with expected healthy contractility and β-AR response. Despite these limitations, these hearts represent normal human physiology as close as currently possible.

## Electronic supplementary material

Supplementary Information
