# Supplementary material for: β-adrenergic stimulation augments transmural dispersion of repolarization via modulation of delayed rectifier currents IKs and IKr in the human ventricle
Source: Sci Rep. 2017 Nov 21;7:15922. doi: 10.1038/s41598-017-16218-3 (PMC5698468; doi:10.1038/s41598-017-16218-3)
Supplement: Supplementary file 1 — Supplementary Information [file 41598_2017_16218_MOESM1_ESM.pdf]

β-adrenergic stimulation augments transmural dispersion of repolarization via modulation of delayed rectifier currents I_{Ks} and I_{Kr} in the human ventricle

Kang C; Badiceanu A, Brennan JA, Gloschat C; Qiao Y; Trayanova N; Efimov IR

Online Supplement

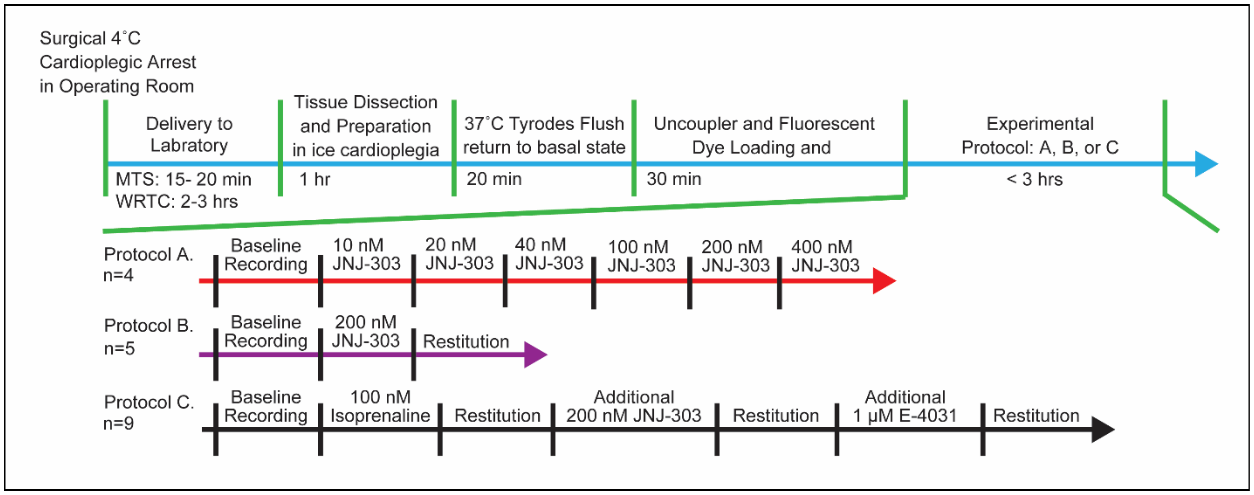

Supplemental Figure 1.

Three experimental protocols were performed from total of 18 donor hearts. Total experimental time was approximately 4-5 hours. While surgical procedure in the operating room and delivery time to the laboratory differed between donor institutions, all other preparation procedures were the same.

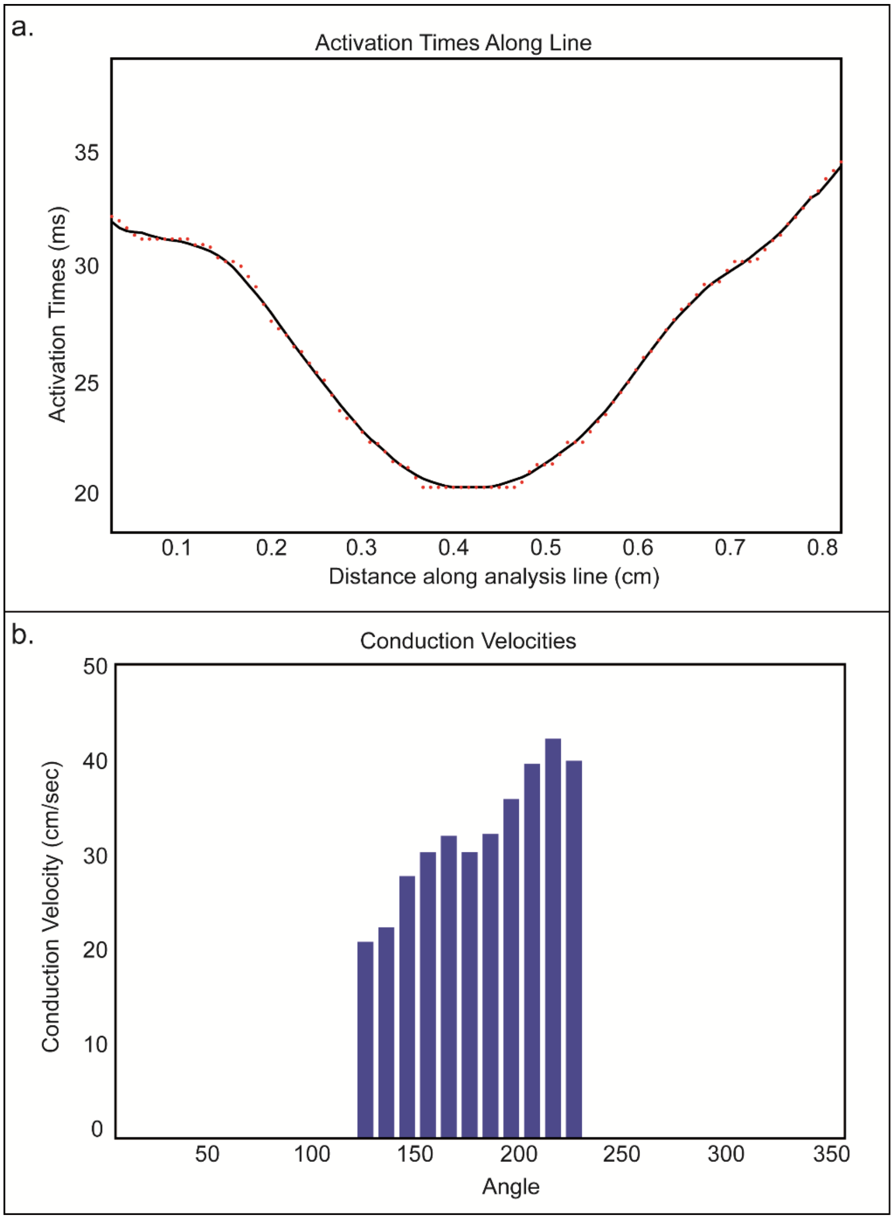

Supplemental Figure 2.

Conduction velocity (CV) was calculated by obtaining activation times (dV/dt_{max}) along a line. By rotating the line around the stimulation site. the minimum CV was set as the transverse direction while the maximum CV was set as the longitudinal direction. These directions were maintained for the duration of the study.

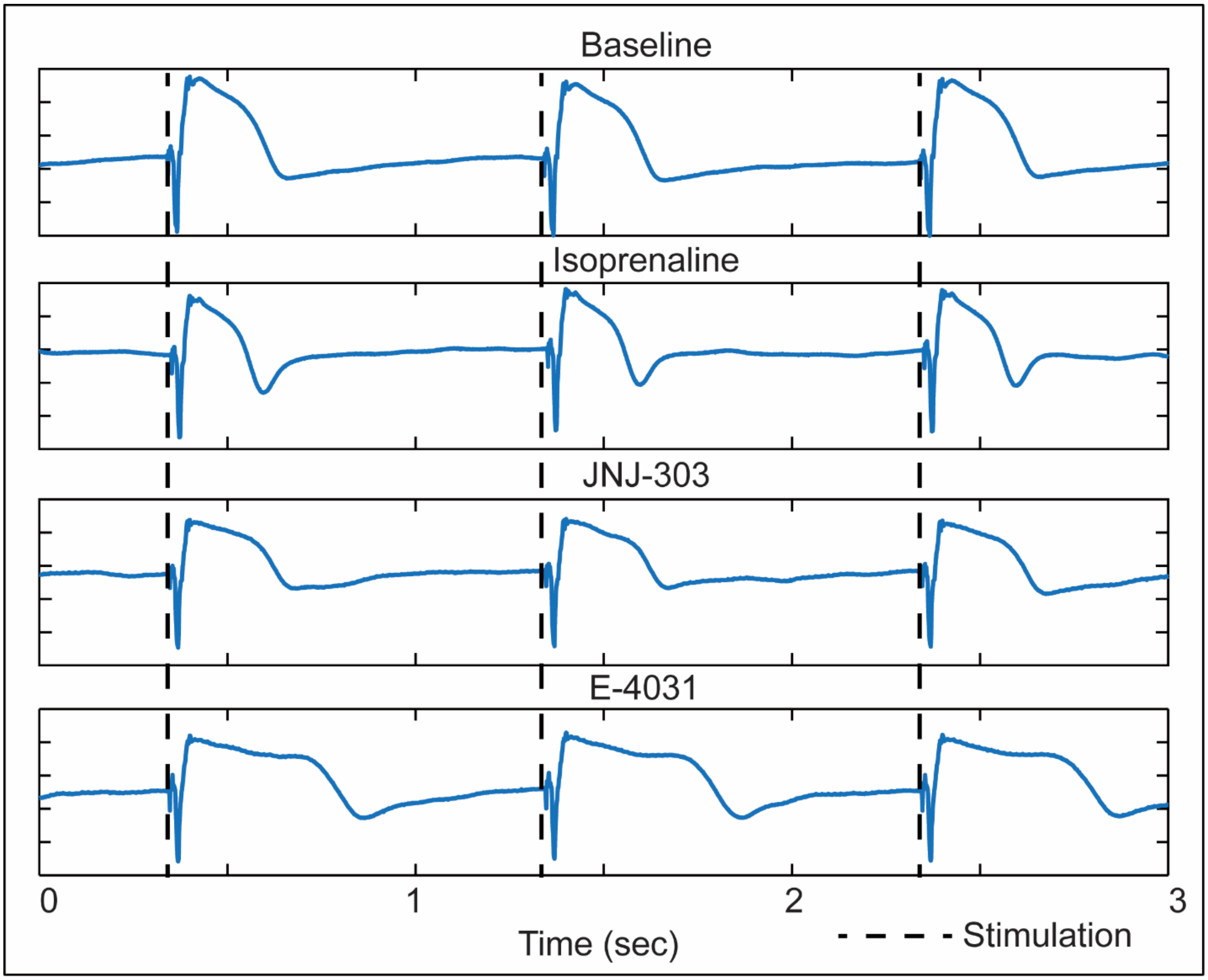

Supplemental Figure 3.

Representative pseudo ECG recordings taken from each condition. The equivalent QRS complex was altered similarly to how action potential was altered. QRS complex was narrowed by isoprenaline but widened with JNJ-303 and E-4031.

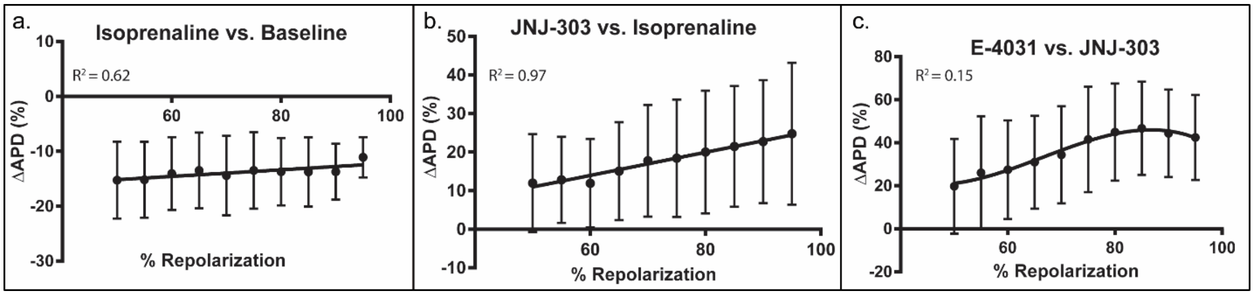

Supplemental Figure 4.

(a, b, c) Fitted curves for all three comparisons indicated that isoprenaline and JNJ-303 affected % ΔAPD80 linearly, while E-4031 was best fitted with an exponential curve.

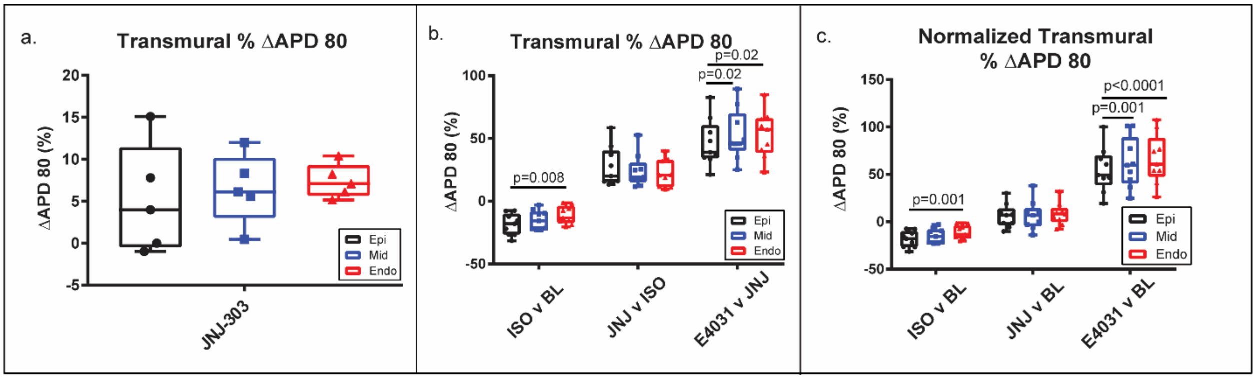

Supplemental Figure 5.

Transmural dispersion as expressed as a percentage to illustrate the difference. Results were very similar to actual ms values in Figure 6.

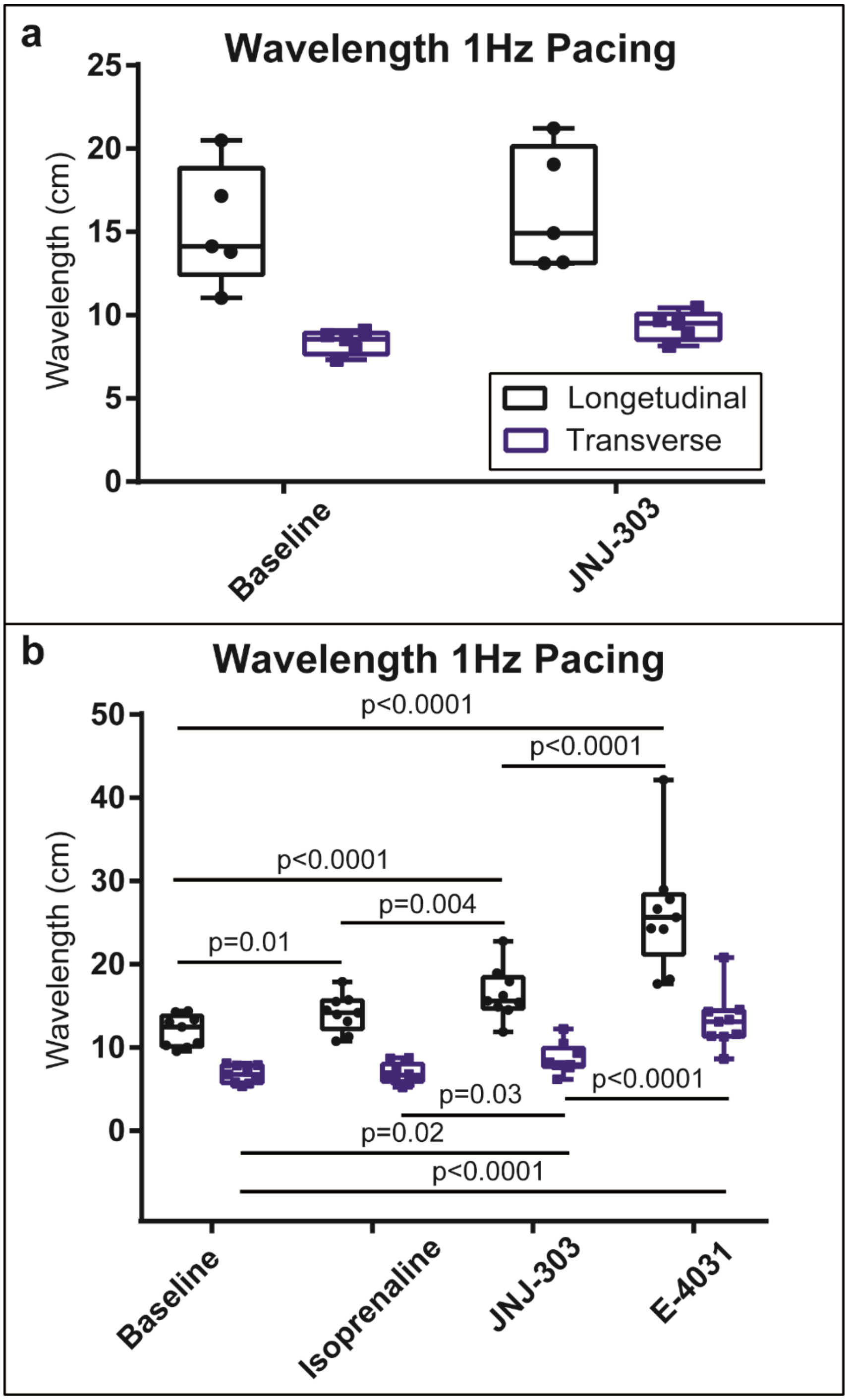

Supplemental Figure 6.

Wavelength of excitability was calculated by $CV_{L/T}$ * APD80. Wavelength was unchanged with isolated JNJ-303. However, wavelength was increased with application of every pharmacologic intervention. This increase in wavelength increased the amount of tissue volume needed to sustain arrhythmia.

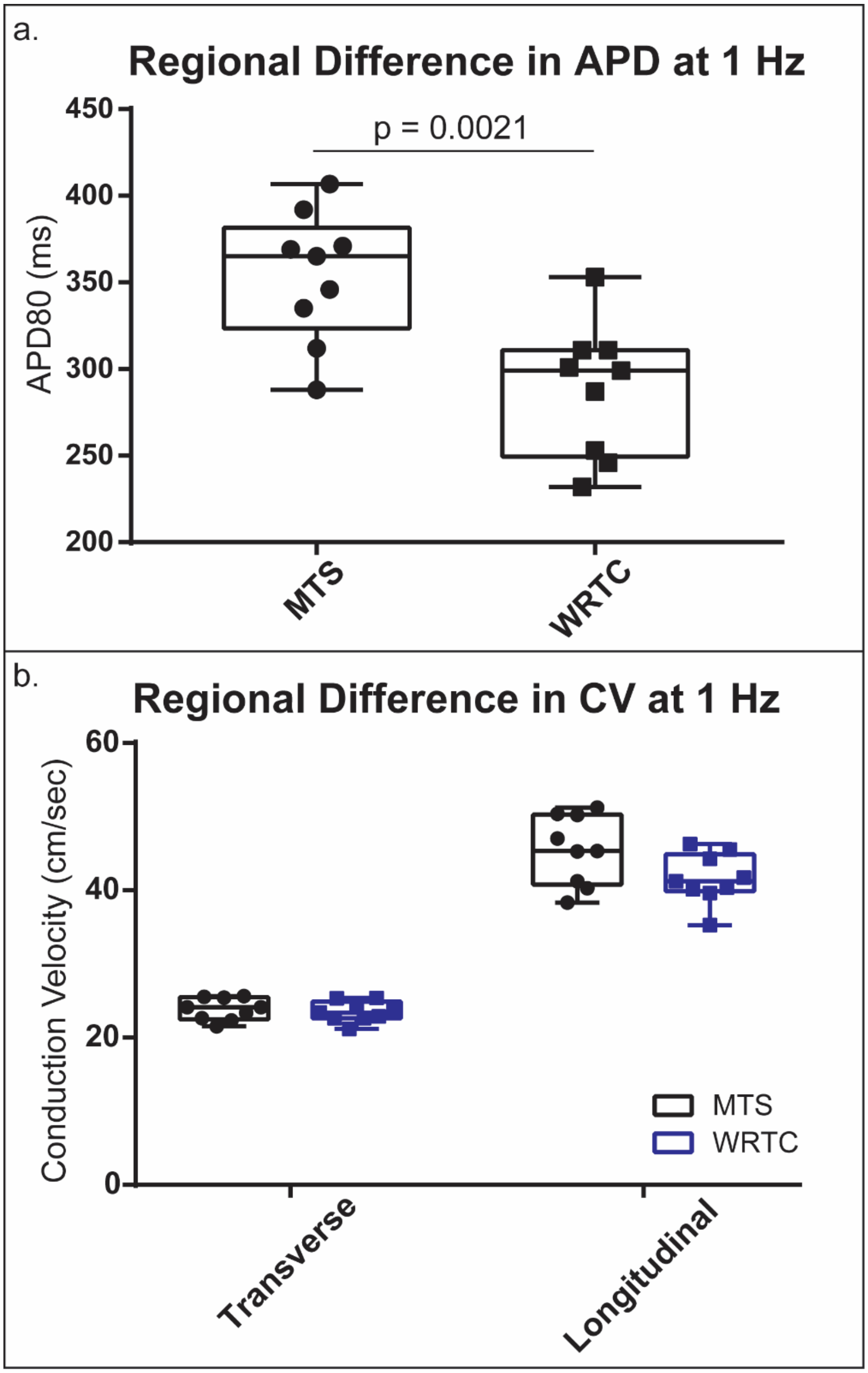

Supplemental Figure 7.

Differences in hearts acquired in St. Louis from MTS compared to hearts acquired in Washington DC from WRTC. APD80 was significantly longer in MTS hearts, while both transverse and longitudinal CV were the same.

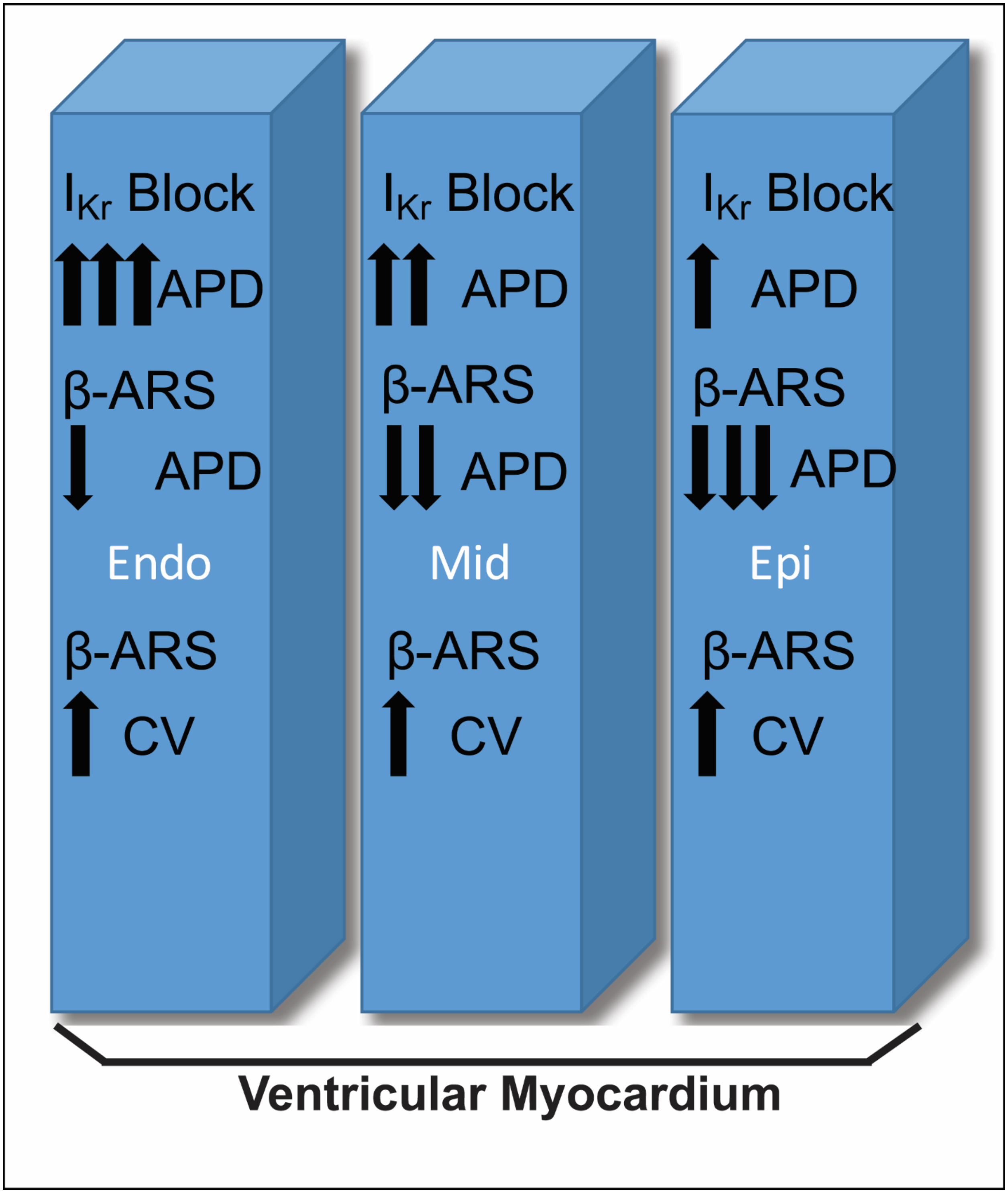

Supplemental Figure 8.

Summary of increased transmural gradient generating substrates for arrhythmia. APD80 gradient was exacerbated from β-AR stimulation and I_{Kr} blockade. Increased CV also played a role in creating functional block in tissue.
